# Rapidly Benchmarking Large Language Models for Diagnosing Comorbid Patients: Comparative Study Leveraging the LLM-as-a-Judge Method

**DOI:** 10.2196/67661

**Published:** 2025-08-29

**Authors:** Peter Sarvari, Zaid Al-fagih

**Affiliations:** 1 Rhazes AI, First Floor, 85 Great Portland Street, London, W1W 7LT, United Kingdom

**Keywords:** large language model, LLM, GPT-4, Gemini, Claude, retrieval-augmented generation, clinical medicine, diagnosis, diagnostic ability of LLMs, artificial intelligence, AI in medicine, AI in health care

## Abstract

**Background:**

On average, 1 in 10 patients die because of a diagnostic error, and medical errors represent the third largest cause of death in the United States. While large language models (LLMs) have been proposed to aid doctors in diagnoses, no research results have been published comparing the diagnostic abilities of many popular LLMs on a large, openly accessible real-patient cohort.

**Objective:**

In this study, we set out to compare the diagnostic ability of 18 LLMs from Google, OpenAI, Meta, Mistral, Cohere, and Anthropic, using 3 prompts, 2 temperature settings, and 1000 randomly selected Medical Information Mart for Intensive Care-IV (MIMIC-IV) hospital admissions. We also explore improving the diagnostic hit rate of GPT-4o 05‐13 with retrieval-augmented generation (RAG) by utilizing reference ranges provided by the American Board of Internal Medicine.

**Methods:**

We evaluated the diagnostic ability of 21 LLMs, using an LLM-as-a-judge approach (an automated, LLM-based evaluation) on MIMIC-IV patient records, which contain final diagnostic codes. For each case, a separate assessor LLM (“judge”) compared the predictor LLM’s diagnostic output to the true diagnoses from the patient record. The assessor determined whether each true diagnosis was inferable from the available data and, if so, whether it was correctly predicted (“hit”) or not (“miss”). Diagnoses not inferable from the patient record were excluded from the hit rate analysis. The reported hit rate was defined as the number of hits divided by the total number of hits and misses. The statistical significance of the differences in model performance was assessed using a pooled *z*-test for proportions.

**Results:**

Gemini 2.5 was the top performer with a hit rate of 97.4% (95% CI 97.0%‐97.8%) as assessed by GPT-4.1, significantly outperforming GPT-4.1, Claude-4 Opus, and Claude Sonnet. However, GPT-4.1 ranked the highest in a separate set of experiments evaluated by GPT-4 Turbo, which tended to be less conservative than GPT-4.1 in its assessments. Significant variation in diagnostic hit rates was observed across different prompts, while changes in temperature generally had little effect. Finally, RAG significantly improved the hit rate of GPT-4o 05‐13 by an average of 0.8% (*P*<.006).

**Conclusions:**

While the results are promising, more diverse datasets and hospital pilots, as well as close collaborations with physicians, are needed to obtain a better understanding of the diagnostic abilities of these models.

## Introduction

### Background

In the United States alone, medical errors are the third largest cause of death [1], and within these errors, diagnostic errors result in the death or permanent disability of 800,000 people each year [2]. Research by The National Academy of Medicine [3] as well as Newman-Toker et al [4] estimated that diagnostic errors are responsible for approximately 10% of patient deaths [3,4] and 6%‐17% of hospital complications [3]. Moreover, 75% of diagnostic errors are cognitive errors [5], which are most commonly caused by premature closure and the failure to consider alternatives after an initial diagnosis has been established. Cognitive errors are also naturally linked to the overload and stress physicians experience, with current burnout rates reaching the highest ever levels recorded [6]. Given the recent progress in artificial intelligence (AI), large language models (LLMs) have been proposed to help with various aspects of clinical work, including diagnosis [7]. GPT-4, an LLM developed by OpenAI, has shown promise in medical applications with its ability to pass medical board exams in multiple countries and languages [8-11].

### Comparing the Diagnostic Abilities of LLMs

Limited studies have attempted to compare the diagnostic abilities of LLMs and have mostly included (1) clinical vignettes; (2) case records directly from clinics; and (3) case reports, such as the *New England Journal of Medicine* (NEJM) Case Challenges. The latter are more complex than clinical vignettes and contain red herrings and other distractors to truly challenge a physician [12]. Khan and O’Sullivan [12] used 10 case challenges and compared diagnoses from GPT-3.5, GPT-4 (Bing), and Gemini 1.5 with the help of 10 physicians who filled out a grading rubric. The authors reported strong agreement among the graders who collectively preferred Gemini among the 3 models. Chiu et al [13] used 102 case records from the Massachusetts General Hospital and showed that GPT-4 outperformed Bard and Claude 2 in terms of diagnostic accuracy based on the *International Classification of Diseases, 10th Revision* (*ICD-10*) hierarchy. Shieh et al [14] asked GPT-3.5 and GPT-4 to analyze 109 USMLE (United States Medical Licensing Examination) Step 2 clinical knowledge practice questions (vignettes) as well as 63 case reports from various journals. The researchers concluded that while GPT-4 was 87.2% accurate on the vignettes, it was only able to create a shortlist of differential diagnoses for 47 of the case reports (75%). Other scholars have assessed the capabilities of various LLMs within a given specialty, such as otolaryngology [15] and radiology [16].

Many authors have focused on evaluating the diagnostic ability of a single LLM: GPT-4 was the most popular choice as it was generally the most accurate LLM at the time. Eriksen et al [17] asked GPT-4 to choose 1 of 6 diagnostic options for each of 38 NEJM case challenges, whereas Kanjee et al [18] relied on NEJM clinicopathological case conferences and tasked GPT-4 to first state the most likely diagnosis and then give a list of differentials. Manual review by the authors concluded that in 45 out of the 70 cases, the correct answer was included in the differentials (in 27 cases, it was the most likely diagnosis). Shea et al [19] used GPT-4 to diagnose 6 patients with extensive investigations but delayed definitive diagnoses and showed that GPT-4 has the potential to outperform clinicians and alternative diagnostic tools such as the Isabel DDx companion. Fabre et al [20] assessed 10 NEJM cases, and while they concluded that the final diagnosis was correctly identified by the AI in 8 cases (it was included in the list of differentials), they also assessed treatment suggestions and found that GPT-4 failed to suggest adequate treatment in 7 cases. Notably, some researchers focused on assessing agreement between doctors and GPT-4, rather than evaluating the accuracy directly. Hirosawa et al [21,22] measured the Cohen κ coefficient in 2 different studies, with the first one relying on cases from the American Journal of Case Reports and the second one primarily relying on 52 complex case reports published by the authors. In both cases, the researchers found fair to good agreement (0.63 [21] and 0.86 [22], respectively) between doctors and GPT-4.

These evaluation strategies work for case challenges but would not suffice for a large cohort of highly comorbid real patients, such as the Medical Information Mart for Intensive Care-IV (MIMIC-IV) [23], where patients might have multiple conditions concurrently. To solve this issue, Sarvari et al [24] outlined a methodology to use AI-assisted evaluation (LLM-as-a-judge [25]) to quickly estimate the diagnostic accuracy of different models on a set of highly comorbid real hospital patients. This automated approach not only allows the evaluation of larger datasets (we increased the sample size 10-fold from <100 [typically seen in evaluations based on clinical cases] to 1000), but also facilitates quick benchmarking of multiple models, which is our goal in this study. Automated evaluation provides reliable estimates, as judged by 3 medical doctors in the aforementioned study [24], and as AI models improve, we only expect this to become better. Moreno and Bitterman [26] also hinted at nonhuman evaluation as a method to allow for a larger-scale beta test, and Zack et al [27] actively employed this method to match generated diagnoses to ground truth diagnoses and shared the prompt as supplementary material.

Despite recent successes, there are subdomains where GPT-4o has been proven to be inferior to alternative AI methods or human diagnosis, particularly when it comes to medical image analysis. GPT-4o was found to perform poorly in detecting pneumonia from pediatric chest x-ray images compared to traditional convolutional neural network–based methods [28]. Zhang et al [29] compared GPT-4o to 3 medical doctors in terms of their abilities to diagnose 26 glaucoma cases and found, using Likert scales, that GPT-4o performed worse than the lowest-scoring doctor in the completeness category. Cai et al [30] assessed the clinical utility of GPT-4o in recognizing abnormal blood cell morphology, an important component of hematologic diagnostics, in 70 images. The LLM achieved an accuracy of only 70% (compared to 95.42% accuracy for hematologists), as reviewed by 2 experts in the field.

### Objective

In this study, we compared the diagnostic abilities of 18 different LLMs from 6 different companies on 1000 electronic patient records, using 3 prompts and 2 temperature settings. Given that the patient records contained the final diagnostic codes of the patients (the ground truth diagnoses), we used the LLM-as-a-judge method, where the assessor LLM merely needs to compare the generated diagnoses from the 18 different LLMs to the ground truth for each of the 1000 patients. We hypothesized that there would be significant differences between the diagnostic abilities of the evaluated LLMs. We also postulated that prompting and hyperparameter (temperature) changes would cause significant differences in the results. Finally, we investigated whether retrieval-augmented generation (RAG) can boost the model’s hit rate by utilizing a reference document [31] that includes the latest clinical reference ranges, offering precise guidance to the model for identifying abnormalities [31].

## Methods

### Ethical Considerations

The MIMIC-IV is a publicly available database and was previously ethically approved by the institutional review boards at Beth Israel Deaconess Medical Center (2001P001699) and the Massachusetts Institute of Technology (0403000206) in accordance with the tenets of the Declaration of Helsinki. The waiver of the requirement for informed consent was included in the institutional review board approval, as all protected health information was deidentified [23]. One of the authors (PS) was granted access to the database after completing training in human research (CITI Human Research certification number: 54889098) and signing a data use agreement in PhysioNet (agreement number 64081). The experiments described in this paper were mostly conducted on Microsoft Azure (Azure OpenAI service), Google Vertex AI, or Anthropic Claude, according to the “Responsible use of MIMIC data with online services like GPT” guidance by PhysioNet [31]. Additionally, the authors relied on the Cohere application programming interface (API) because the Cohere models were not available on any of the other platforms. This was deemed safe given that Cohere is Health Insurance Portability and Accountability Act compliant, stores the data on Google Cloud, and does not use the data for model training (once the user has opted out) [32]. Occasionally, the direct OpenAI API connection was used after ensuring that no data (including input, output, and user feedback) were shared with OpenAI. The code associated with this publication has been shared in an open repository, and information is provided in the *Data Availability* section of this manuscript.

### LLM Setup and Evaluation

The models we compared for medical diagnosis in our analysis are summarized in Table 1.

**Table 1.** List of the 21 models compared in this study.

Model	Date/version used	Platform	Reference
GPT-4-Turbo-preview	November 6, 2023	Microsoft Azure API^a^	[33]
Medlm-medium	May 8, 2024, and March 19, 2025	Google Vertex AI^b^ API	[34,35]
Gemini-1.5-Pro-preview	April 9, 2024	Google Vertex AI API	[36]
Command R Plus	April 2024	Cohere API	[37]
GPT-4o	May 13, 2024	Microsoft Azure API	[38]
Claude-3‐5-Sonnet	June 20, 2024	Anthropic Claude API	[39]
GPT-4o	August 6, 2024	Microsoft Azure API	[38]
Mistral-large	August 22, 2024	Microsoft Azure API	[40]
Meta-Llama-3.1-405B-Instruct	August 22, 2024	Microsoft Azure API	[41]
GPT-4o	November 20, 2024	Microsoft Azure API	[38]
o3-mini	January 31, 2025	Microsoft Azure API	[42]
Claude-3‐7-Sonnet	February 19, 2025	Anthropic Claude API	[43]
GPT-4.5-preview	February 27, 2025	OpenAI API	[44]
Gemini-2.0-Flash	March 19, 2025	Google Vertex AI API	[36]
Llama-4-Scout-17b-16e	April 5, 2025	Google Vertex AI API	[45]
GPT-4.1	April 14, 2025	Microsoft Azure API	[46]
o3	April 16, 2025	Microsoft Azure API	[47]
o4-mini	April 16, 2025	Microsoft Azure API	[48]
Claude-Sonnet-4	May 14, 2025	Anthropic Claude API	[49]
Claude-Opus-4	May 14, 2025	Anthropic Claude API	[50]
Gemini-2.5-Flash	June 17, 2025	Google Vertex AI API	[36]

a API: application programming interface.

b AI: artificial intelligence.

The automated evaluation was performed by GPT-4‐1106-preview (GPT-4 Turbo) and by GPT-4.1 (April 14, 2025) via OpenAI API with always zero temperature.

**Figure 1.**
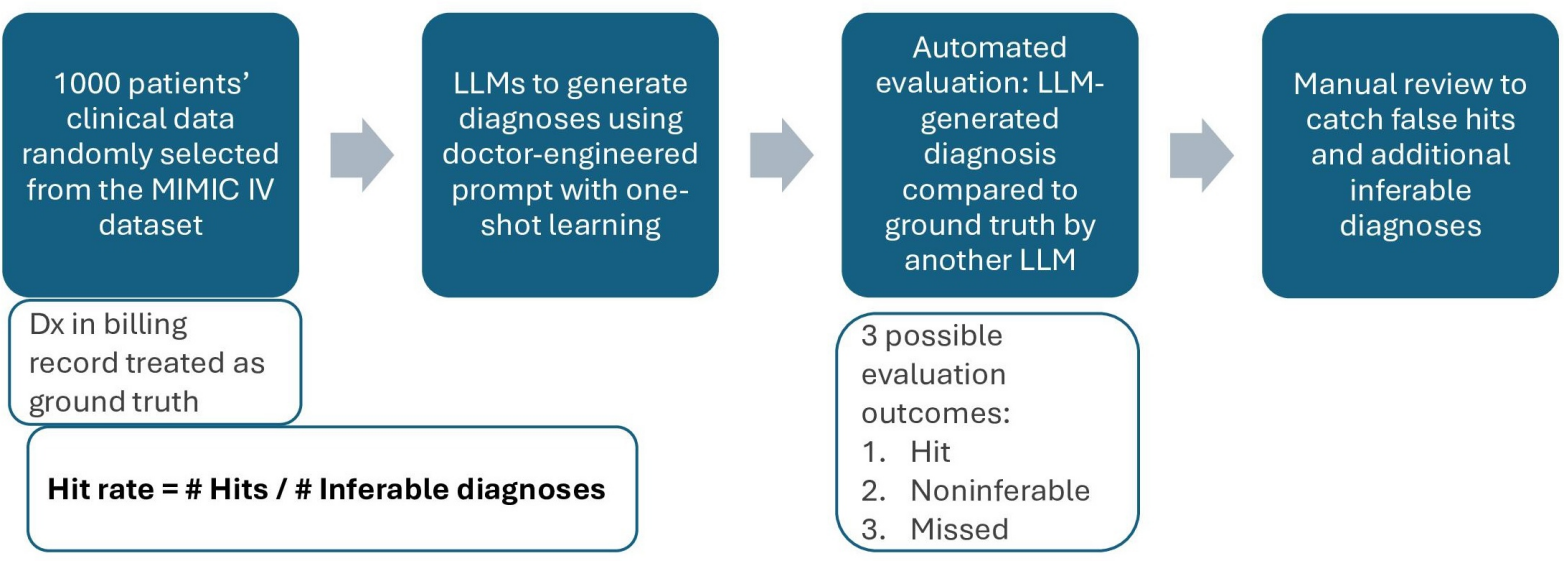
Summary of the evaluation methodology. Dx: diagnosis; LLM: large language model; MIMIC IV: Medical Information Mart for Intensive Care-IV.

The MIMIC-IV data sample containing 1000 hospital admissions (median number of words: 694, IQR 329; cap at 1000) and diagnostic and evaluation prompts were taken from [24]. The evaluation methodology is summarized in Figure 1. In some experiments, the total number of diagnoses (hits + misses + noninferables + exclusions) was slightly lower than the expected number of 14,403 due to diagnostic or self-evaluation glitches (LLMs refused to answer or did not follow the requested format exactly). We marked the experiment valid if these errors accounted for <0.25% of the total ground truth diagnoses; otherwise, we reran the failed responses until the experiment became valid.

Our initial idea was to simply compare the predicted *International Classification of Diseases* (*ICD*) codes to the *ICD* codes extracted from the patients’ billing reports (ground truth) and examine what proportion was guessed correctly. However, the MIMIC-IV data did not contain patient history (previous diagnoses and medications), patient physical examinations, and other useful measurements such as electrocardiography (ECG). Of course, without medication records, we would not know if the patient has a coagulation disorder or is taking anticoagulants, and without ECG data, we would not be able to diagnose atrial fibrillation. Hence, such diagnoses were not inferable from the data, and we excluded them. Further, given the lack of patient diagnostic history and the very specific *ICD* code names, it would not be possible to distinguish between diseases with different onsets (acute vs chronic) or between diseases with differing degrees of severity. Hence, we deem the prediction correct if the predicted and ground truth diagnoses are 2 related diseases (eg, caused by the same pathogen and affecting the same organ), which are indistinguishable given the patient data. In this case, the further tests the LLM has been instructed to suggest in the prompt from Sarvari et al [24] would be of crucial importance to understand the exact disease pathology. There are also *ICD* codes that do not correspond to diagnoses (eg, do not resuscitate, homelessness, and unemployment), and we excluded such codes from this study. We defined a correct prediction as a “hit” and the failure to predict a ground truth diagnosis as a “miss.”

In terms of the evaluation metrics, we solely focused on the hit rate (also called recall, true positive rate, and sensitivity) in this study. The reported hit rate was the average across all the ground truth diagnoses of the 1000 sample patients. The rationale is as follows: for every single disease in the world, the patient may have it or not have it. As such, when making predictions, the LLM is effectively executing binary classifications for every single disease. Of course, even a highly comorbid patient will not have 99.99%+ of the possible diseases, and hence, the metrics related to negative selected elements, such as specificity, are very close to 1 by default and are not meaningful to report. As a result, the meaningful metrics here are precision and hit rate. However, a good quantification of precision is challenging in this case because false positives are difficult to establish, as not every single medical condition ends up on the billing report of the patient. Hence, it is unclear and subjective whether certain well-reasoned diagnostic predictions should be marked as false positives just because they did not show up on the patient’s billing report. As a solution, we have reported the hit rate while (1) indirectly constraining the number of predictions by limiting the LLM output tokens to 4096 and (2) ensuring explainability by asking the LLM to reason why it predicted certain conditions.

### RAG Setup

GPT-4o-05‐13 with RAG was implemented via the Azure Search API. A critical element of RAG is the reference document, from which relevant information is retrieved and supplied to the LLM to enhance its performance. Ideally, such a document should contain key information related to the task at hand, especially details that the LLM may not already know or for which its internal knowledge could be outdated or conflicted. Based on the most frequent diagnostic misses identified in the study by Sarvari et al [24], including anemia, hypoxemia, hypoosmolality, and hypernatremia, we recognized that many of these conditions can be diagnosed primarily through the interpretation of laboratory values against established reference ranges. To address this, we identified the need for clinical guidelines that directly support diagnoses reliant on specific laboratory thresholds. Therefore, we selected a document with laboratory test reference ranges as the reference document for RAG, as these ranges provide explicit criteria needed for accurate identification of such conditions. Accordingly, a document containing laboratory test reference ranges from The American Board of Internal Medicine updated January 2025 [51] was vectorized (embedded by the *text-embedding-3-large* model from OpenAI) and indexed to be used for RAG, with an overlap of 100 tokens and a chunk size of 800 tokens. The 10 closest matching chunks to the patient data input (out of the 32 total chunks, corresponding to over 3 times input token cost reduction) were retrieved using the HNSW algorithm with a bidirectional link count of 4, an efConstruction of 400, an efSearch of 500, and cosine similarity. Note that when we compared the RAG-based diagnostic engine to its non-RAG equivalent, we also leveraged RAG in the automated evaluation. This was to ensure that not just the diagnostic but also the evaluator LLM is aware of the latest clinical reference ranges. This was a crucial step, as *without* explicitly giving the reference ranges to the assessor model, we did not notice a statistically significant improvement caused by RAG.

### Prompt Engineering and Temperature

In this study, we experimented with 3 diagnostic prompts. Prompt A was a highly specific one-shot learning prompt. Prompt A/2 was almost the same as prompt A, but was a bit ambiguous in its way of asking to report the diagnoses. Prompt B provided a detailed background and task description and aimed to help the LLM with organizing its thoughts. The prompts are presented in Textbox 1.

Textbox 1. **Prompts.**

**Prompt A**

“Suggest as many potential diagnoses as possible from the following patient data.

In addition, include previously diagnosed conditions and information about patient’s medical history (if any).

Give exact numbers and/or text quotes from the data that made you think of each of the diagnoses and, if necessary, give further tests that could confirm the diagnosis.

Once you're done, suggest further, more complex diseases that may be ongoing based on the existing diagnoses you already made.

Use the International Classification of Disease (ICD) naming standard for reporting the diagnoses, but you don't have to specify the codes.

Before finalizing your answer check if you haven’t missed any abnormal data points and hence any diagnoses that could be made based on them. If you did, add them to your list of diagnoses.”

The prompt also contains a very detailed example, which can be viewed in the GitHub repository (details are provided in the *Data Availability* section).

**Prompt A/2**

Same as prompt A (including the example), but we asked the model to report the diagnoses in the following (slightly ambiguous) way:

“Use the International Classification of Disease (ICD) standard for reporting the diagnoses.”

**Prompt B**

“You are an expert diagnostician machine for use by doctors. If the user input is not patient data, you politely decline the request. Please suggest diagnoses and conditions, followed by the evidence points supporting each diagnosis in the form of bullet points. Include previous diagnoses and pertinent information about the patient’s medical history (if any). Pay close attention to all the history and investigations provided. Put asterisks around the diagnoses to highlight them. Give each evidence points as a separate bullet point beneath the diagnosis. Include in your evidence points any relevant clinical scores that can be calculated from the information I have given. Do not explain the evidence points, only state them. For every diagnosis you list, if there are alternative differentials possible, state the most likely three in a bullet point beneath the evidence points (you do not need to state the evidence supporting them - you only need to do that for the main diagnoses). For the main diagnoses, give only confirmed diagnoses and evidence points that can be inferred solely based on the information I have given - do not use any other information. Only give me the information I have asked for - do not give me any other information. Do not give me any introductions or conclusions, safety instructions, or safety warnings. Use British English.

To illustrate how the information should be presented:

*MAIN DIAGNOSIS 1 AS HEADING*

evidence points to support MAIN DIAGNOSIS 1

The final bullet point is alternative differentials to consider: alternative 1, alternative 2, alternative 3

*MAIN DIAGNOSIS 2 AS HEADING*

evidence points to support MAIN DIAGNOSIS 2

The final bullet point is alternative differentials to consider: alternative 1, alternative 2, alternative 3

and so on...

Before finalizing your answer check if you haven’t missed any abnormal data points and hence any diagnoses or alternative differentials that could be made based on them. If you did, add them to your reply. If two diagnoses are commonly caused by the same underlying disease, have them under one header, which is the underlying disease.”

**Added prompt section for retrieval-augmented generation (both for diagnosis and auto-evaluation)**

This system is Retrieval-Augmented Generation (RAG) enabled. **Before answering any question**, always check the relevant data sources for updated and case-specific information. Ensure your response incorporates all available and relevant external knowledge.

Apart from the prompts, we also experimented with 2 different hyperparameter values, namely the default temperature (0.7 or 1, depending on the model) and a temperature of zero. In the *Results* section, we report outcomes for all prompts and temperature values measured, and test whether they statistically significantly influence the hit rate. For the prompt used for the automated evaluation, please see the study by Sarvari et al [24].

### Hypothesis Testing

To compare whether the hit rates (proportions) of 2 different models are statistically significant, we used the pooled *z*-test, which can be performed even when the number of inferable diagnoses slightly differs between 2 experiments. We chose pooling because our null hypothesis involves testing equal proportions, implying the same true proportion of success, *p* (which also means equal variances, since each of the proportions follows a binomial distribution). We chose the *z*-test because the sampling distribution of the sample mean (the number of correctly identified diagnoses) follows a normal distribution as the sample size increases, according to the Central Limit Theorem. We used a 2-sided test, unless otherwise stated. A common rule of thumb regarding the Central Limit Theorem for proportions is to require both *np* and *n(1-p*) to be larger than 10 (in other words, have at least 10 correctly and 10 incorrectly identified diagnoses). This requirement was easily satisfied in our case. Finally, we calculated the 95% CI of the hit rate by adding and subtracting 1.96 (*z*_0.05_) times the standard error of the mean, which is simply the square root of *p(1-p)/n*. To make the statistical significance calculations manageable, we assumed, during the calculations, that the automated evaluation would make no mistakes.

## Results

### LLM Evaluation With GPT-4 Turbo and Multiple Prompts

The 1000 randomly selected patients were highly comorbid, with an average of 14.4 (IQR 10; minimum: 1, maximum: 39) distinct diagnostic codes per patient. Table 2 shows the results of all the GPT-4 Turbo evaluation experiments we ran in this study. The best overall hit rate of 99.8% (rounded to the first decimal point) was achieved by the GPT-4.1 foundation LLM with prompt B and default temperature. The next best hit rate of 99.7% was achieved by GPT-4o 11‐20 with prompt B and zero temperature, Claude 3.7 with prompt B and default temperature, and GPT-4.5 with both default and zero temperature settings.

**Table 2.** Results of all GPT-4 Turbo evaluation experiments.

Company and model		Prompt A hit rate (%)		Prompt B hit rate (%)	
		Zero temperature	Default temperature	Zero temperature	Default temperature
Google					
MedLM (medlm-medium)		—^a^	98.7^b^*;* 92.9	—	—
Gemini 1.5 Pro (preview-0409)		98.8	97.7	—	—
Gemini 2 Flash		—	98.3	99.4	99.6
Meta					
Llama 3.1		—	98.8	—	—
Mistral					
Mistral 2 Large		—	99.1	—	—
Cohere					
Command R Plus (04‐2024)		—	99.3	99.0	98.9
Anthropic					
Claude 3.5 (Sonnet-20240620)		99.5	98.8	—	—
Claude 3.7 (Sonnet-20250219)		—	99.2	99.6	99.7
OpenAI					
GPT-4 11‐06-preview (Turbo)		—	99.3^b^; 99.0	—	99.3
o3-mini (2025-01-31)		—	99.3^b^; 99.3	—	98.7
GPT-4o 05‐13		99.2; 99.4; 99.5	98.6^b^; 99.4	99.4	99.3; 99.3; 99.3; 99.5
GPT-4o 08‐06		98.2^b^; 99.0	97.8^b^; 99.1	99.3; 99.3	99.3
GPT-4o 11‐20		—	98.4^b^; 99.1	99.7	99.6
GPT-4.5 (preview 2025-02-27)		—	98.8^b^; 99.3	99.7	99.7
GPT-4.1		—	—	—	99.8

a Not applicable. No experiments run with such settings.

b Prompt A/2 result.

In Table 3, we have included further details for the best results (hit rate of at least 99.5%) in the GPT-4 Turbo evaluation experiments.

**Table 3.** Details of the best GPT-4 Turbo evaluation experiments (diagnostic hit rate of at least 99.5%).

Model	Settings^a^	Hit rate (%; hits/inferable), mean (SD)	Hits, n	Hits + misses (inferable), n	Noninferable + excluded, n	Link to result
Claude 3.5 (Sonnet-20240620)	Prompt A, T=0	99.5 (0.2)	7054	7089	7311	[52]
GPT-4o 05‐13	Prompt A, T=0	99.5 (0.2)	7259	7296	7017	[53]
GPT-4o 05‐13	Prompt B, default T	99.5 (0.2)	6802	6835	7567	[54]
Gemini 2 Flash	Prompt B, default T	99.6 (0.2)	6761	6790	7612	[55]
Claude 3.7 (Sonnet-20250219)	Prompt B, T=0	99.6 (0.2)	6761	6790	7612	[56]
GPT-4o 11‐20	Prompt B, default T	99.6 (0.2)	6953	6980	7392	[57]
GPT-4o 11‐20	Prompt B, T=0	99.7 (0.1)	6838	6860	7512	[58]
Claude 3.7 (Sonnet-20250219)	Prompt B, default T	99.7 (0.1)	6862	6885	7518	[59]
GPT-4.5 (preview 2025-02-27)	Prompt B, default T	99.7 (0.1)	7014	7036	7367	[60]
GPT-4.5 (preview 2025-02-27)	Prompt B, T=0	99.7 (0.1)	6897	6917	7484	[61]
GPT-4.1	Prompt B, default T	99.8 (0.1)	7229	7246	7157	[62]

a T indicates temperature.

### LLM Evaluation With GPT-4.1

Given that GPT-4.1 was the top-performing diagnostic LLM when evaluated by GPT-4 Turbo, we postulated that the automated evaluation quality would increase if we used this model as the evaluator. Table 4 shows the details of the GPT-4.1 evaluation experiments.

**Table 4.** Details of all GPT-4.1 evaluation experiments (prompt B, default temperature).

Model	Hit rate (%; hits/inferable), mean or mean (SD)	Hits, n	Hits + misses (inferable), n	Noninferable + excluded, n	*P* value^a^	Link to result
o4-mini	91.8 (0.8)	4630	5045	9358	—^b^	[63]
GPT-4o 05‐13 total	93.0 (0.4)	15,105	16,240	26,969	.003	
GPT-4o 05‐13 run0	93.0	5008	5386	9017	—	[64]
GPT-4o 05‐13 run1	93.1	5061	5436	8967	—	[65]
GPT-4o 05‐13 run2	92.9	5036	5418	8985	—	[66]
LLaMa4 Scout	93.4 (0.7)	5113	5472	8931	.28	[67]
Claude 4 Sonnet	94.4 (0.6)	5030	5327	9076	.03	[68]
Claude 4 Opus	95.2 (0.6)	5061	5317	9086	.08	[69]
o3-mini	96.6 (0.5)	5348	5534	8896	<.001	[70]
GPT-4.1	96.8 (0.5)	5394	5575	8828	.74	[71]
Gemini 2.5	97.4 (0.4)	5767	5921	8482	.04	[72]

a Significance (to previous row).

b Not applicable.

The top-performing model Gemini 2.5 found the exact condition or one deemed directly related to it (ie, equally reasonable to infer given the patient data) in 5767 cases out of the 5921 inferable diagnoses, giving it a diagnostic hit rate (sensitivity) of 97.4% (95% CI 97.0%‐97.8%). The 5767 hits the model made corresponded to 1409 unique diagnoses. Table 5 summarizes the most common hits and misses by this LLM.

**Table 5.** Most common diagnostic hits and misses made by Gemini 2.5 (prompt B, temperature=1).

Disease	Type	Occurrence, n
Heart failure	Hit	378
Anemia	Hit	338
Diabetes mellitus	Hit	315
Kidney failure	Hit	223
Acidosis	Hit	151
Hypokalemia	Miss	16
Hypotension	Miss	8
Dehydration	Miss	7
Hypoxemia	Miss	7
Hyperkalemia	Miss	6
Hypo-osmolarity and hyponatremia	Miss	6

By comparing Table 3 with Table 4, we can observe that GPT-4.1, in general, classified fewer diagnoses as hits and more as noninferables. In Table 6, we contrast a few cases where GPT-4 Turbo hits were shown as noninferables by GPT-4.1, to gain insights into how the different models interpret the auto-evaluation task.

**Table 6.** Contrast of cases between GPT-4 Turbo and GPT-4.1.

Hadm ID	Diagnosis	GPT-4 Turbo evaluation (question 1^a^)	GPT-4.1 evaluation (question 1 response is “yes”; question 2^b^)
22780338	Unspecified asthma with (acute) exacerbation	No, this could be considered as part of the differential diagnosis for *Chronic Pulmonary Disease*	No, there is no mention of a history of asthma, wheeze, reversible airway obstruction, or bronchodilator response in the data provided. Additional data such as a history of asthma, clinical wheeze, or spirometry would have been helpful.
22780338	Body mass index (BMI) 40.0‐44.9, adult	No, this is related to *Chronic Type 2 Respiratory Failure (Hypercapnic Respiratory Failure)* as severe obesity hypoventilation was considered [as an alternative]	No, BMI is not provided in the data. Height and weight or BMI value would have been needed.
22780338	Long-term (current) use of insulin	No, this is related to the management of *Poor Glycemic Control (Diabetes Mellitus, Uncontrolled)*	No, there is no mention of insulin use in the data provided. Medication history would have been needed.
20760364	Displaced bicondylar fracture of right tibia, initial encounter for closed fracture	No, this diagnosis is a more specific version of *Tibial plateau fracture with hemarthrosis (right knee)*	No, the CT^c^ scan describes a “minimally displaced right posterolateral tibial plateau fracture” but does not mention a bicondylar fracture or displacement of both condyles. Additional imaging details or radiology report specifying bicondylar involvement would have been helpful.
22922413	Ventricular tachycardia	No, this could be related to *Volume Overload / Mild Heart Failure / Congestion* as cardiac arrhythmias can be a complication of heart failure.	No, there is no ECG^d^ or rhythm data provided in the initial query to directly infer ventricular tachycardia. Additional data such as telemetry, ECG, or clinical documentation of arrhythmia would have been helpful.
27531305	Delirium due to a known physiological condition	No, this is similar to the increased confusion mentioned in the postoperative state and could be a complication of the perioperative period. [mentioned as an alternative]	No, the initial data mentions “increased confusion” in the imaging indication, but there is no formal cognitive assessment, no details on mental status changes, or other features of delirium (eg, inattention, fluctuating course, disorganized thinking). Additional data such as a formal mental status exam, CAM (Confusion Assessment Method) score, or documentation of acute onset and fluctuating course would have been helpful.

a Question 1 asks if this is a new diagnosis; see Sarvari et al [24] for the evaluation prompt.

b Question 2 asks if the new diagnosis could have been inferred from the data; see Sarvari et al [24] for the evaluation prompt.

c CT: computed tomography.

d ECG: electrocardiography.

Note that evaluation with GPT-4.1 appeared generally more aligned with the intended purpose of the evaluation prompt, and while in some cases there was no obvious right or wrong answer, a stricter, more careful evaluation is generally preferred. This and other GPT-4 Turbo evaluation shortcomings are discussed in the *Limitations* section.

### Prompt Engineering and Temperature

Comparing prompt A/2, prompt A, and prompt B, we observed that, compared to GPT-4 05‐13, the newer, larger models (Gemini 2, Claude 3.7, GPT-4o 08‐06, GPT-4o 11‐20, and GPT-4.5) preferred prompt B over prompt A, and prompt A over prompt A/2 (where measured), while older or smaller models (MedLM, Command R Plus 04‐2024, GPT-4 Turbo, and GPT-o3-mini) did not show such clear patterns, with many seeming to have the opposite preference. These findings are summarized in Table 7 and Table 8.

**Table 7.** Prompt preference of the latest models.

Model^a^	Prompt A/2 preference, % (n/N)		Prompt A preference, % (n/N)	Prompt B preference, % (n/N)	*P* value
Gemini 2 Flash	—^b^		98.3 (6340/6450)	99.6 (6761/6790)	<.001^c^
Claude 3.7 (Sonnet-20250219)	—		99.2 (6784/6840)	99.7 (6862/6885)	<.001^c^
GPT-4o 08‐06; T=0	98.2 (6325/6440)		99.0 (7014/7083)	99.3 (13,150/13,248)	<.001^d^; .08^c^
GPT-4o 08‐06; default T	97.8 (6321/6462)		99.1 (7115/7184)	99.3 (6733/6781)	<.001^d^; .10^c^
GPT-4o 11‐20	98.4 (6736/6846)		99.1 (7326/7390)	99.6 (6953/6980)	<.001^c^^,^^d^
GPT-4.5 (preview 2025-02-27)	98.8 (6761/6844)		99.3 (7057/7106)	99.7 (7014/7036)	.001^d^; .002^c^

a T indicates temperature.

b Not applicable.

c Prompt A vs prompt B.

d Prompt A/2 vs prompt A.

**Table 8.** Prompt preference of the older or smaller models.

Model	Prompt A/2 preference, % (n/N)	Prompt A preference, % (n/N)	Prompt B preference, % (n/N)	*P* value
MedLM (medlm-medium)	98.7 (6448/6534)	92.9 (5612/6038)	—^a^	<.001^b^
Command R Plus (04‐2024)	—	99.3 (7390/7439)	98.9 (6809/6886)	.003^c^
GPT-4 11‐06-preview (Turbo)	99.3 (6844/6893)	99.0 (6646/6713)	99.3 (6838/6889)	.07^b^; .11^c^
GPT-o3-mini	99.3 (7119/7169)	99.3 (7041/7090)	98.7 (6404/6488)	.96^b^; <.001

a Not applicable.

b Prompt A/2 vs prompt A.

c Prompt A vs prompt B.

During our experiments, for most models, we found no proof for significant differences between zero and default temperatures. However, in the case of Claude 3.5 and prompt A, temperature zero significantly increased performance (*P*<.001).

### RAG Evaluation

We hypothesized that RAG on recently published (2025 January) reference ranges [51] would help LLMs give more accurate and up-to-date diagnoses. To prove this, we chose GPT-4o 05‐13, a fairly accurate model (as shown in Table 3), which had its knowledge cutoff back in October 2023. We ran 6 experiments in total, all using prompt B and default temperature, and all evaluated by RAG-based GPT-4.1 (using the same reference ranges). The results of the 6 experiments are shown in Table 9. The RAG-based model predictions were found to be significantly better than the non-RAG predictions (mean hit rate 92.5% vs 91.7%; *P*<.006). Note that the same (non-RAG) GPT-4o 05‐13 predictions were used for both Tables4  9, which means that the difference in hit rates comes from the difference in evaluation (RAG-based). As expected, giving the assessor model access to the latest reference ranges made it stricter, resulting in a lower estimated hit rate for GPT-4o 05‐13 (93.0% vs 91.7%; mean of 3 runs).

**Table 9.** Retrieval-augmented generation hypothesis results.

Experiment run	GPT-4o 05‐13 hit rate, % (n/N)	GPT-4o 05‐13 RAG^a^ hit rate, % (n/N)
Run 0	91.7 (4961/5411)	92.5 (5119/5533)
Run 1	91.8 (5008/5457)	92.2 (5069/5500)
Run 2	91.6 (4997/5453)	92.7 (5082/5484)
Total	91.7 (14,966/16,321)	92.5 (15,270/16,517)

a RAG: retrieval-augmented generation.

## Discussion

### LLM Diagnoses

In this paper, we compared the diagnostic abilities of multiple LLMs on a subset of the MIMIC-IV dataset, using a previously established LLM-as-a-judge method. The method uses the *ICD* codes from the patient record as the ground truth and (1) removes noninferable diagnoses and (2) accepts similar *ICD* diagnoses as correct predictions when there is not enough data to infer the exact code. We found that Gemini 2.5 was the top-performing LLM with a hit rate of 97.4%, significantly outperforming GPT-4.1 as well as Claude-4 Opus and Sonnet, as evaluated by GPT-4.1. Using automated evaluation via GPT-4 Turbo, we observed that open-source models, such as Mistral 2 and Llama 3.1, performed reasonably well, with performance being better than that of some of the closed-source models from Google but significantly worse than that of alternatives from Anthropic and OpenAI. We also showed that differences in prompting and hyperparameter (temperature) changes can cause significant variations in the results. It was particularly interesting to observe the prompt preferences among the various models tested in the experiments. The latest models demonstrated enhanced knowledge, larger context windows, and greater overall intelligence. Consequently, providing an example (one-shot learning), as seen in prompt A, is not always necessary for these models. However, the precision of the query (with prompt B being more specific than prompt A, which in turn surpasses prompt A/2) appears to be indispensable. Without a clear, well-crafted query, these models may underperform, even compared to their older, smaller counterparts. This highlights the continued importance of prompt engineering, even as models advance. Finally, we concluded that RAG can significantly improve the hit rate of GPT-4o (*P*<.006), confirming our hypothesis that RAG can enhance LLM performance. We hypothesized that RAG using clinical reference ranges would help the LLM have fewer diagnostic misses for conditions where the reference document explicitly provides up-to-date normal clinical values. This improvement may occur because the document either supplies new or updated information not memorized by the LLM during training or directly “reminds” the LLM of the correct reference ranges at inference time. To illustrate, we compared the hit rates for all osmolality-related conditions in the dataset (hyper- or hypo-osmolality, potentially with hypo- or hypernatremia) between the RAG architecture and baseline GPT-4o. Although the RAG-supported LLM with access to the American Board of Internal Medicine document of laboratory reference ranges [51], which clearly highlighted normal osmolality values, still failed to diagnose many abnormal osmolality-related conditions, it correctly identified more cases than its non-RAG counterpart (163/228 vs 146/226). Although this difference was not statistically significant (*P*=.058), the trend supports the utility of RAG for these types of diagnoses. It is important to note, however, that it may not be technically feasible for the RAG system to identify all possible diagnoses in every case. One reason is that the LLM in the RAG architecture only receives the 10 most relevant chunks of the reference document (based on cosine similarity) out of a total of 32 chunks. As a result, depending on the patient data, some relevant reference ranges, such as those for sodium or osmolality, may not be included in the information passed to the LLM for a given case.

### LLM Evaluation

Regarding the evaluation of diagnostic predictions, other researchers have used *ICD* chapters [13], as well as 515 Clinical Classifications Software Refined (CCSR) categories and 22 CCSR bodies [73], to compare the diagnostic predictions to the ground truth and have reported accuracies at these different levels. While this method is very helpful for creating a fast and objective evaluation framework, it does not consider whether the data available are enough to arrive at the ground truth diagnosis (or to a similar one within the same CCSR category), resulting in a more conservative reported diagnostic accuracy. In other words, by using this method, one assumes that the information in the data used (MIMIC-III in the study by Shah-Mohammadi and Finkelstein [73]) is sufficient to make the reported *ICD* diagnoses. In addition, a major drawback of attempting to predict *ICD* chapters and CCSR categories is that 2 physiologically very different diseases may end up in the same category. For example, “Type 1 diabetes mellitus without complications” (*ICD-10* code: E109) and “Type 2 diabetes mellitus without complications” (*ICD-10* code: E119) belong to the same CCSR category 1 of *END002*. This means that if the LLM predicted type 1 diabetes, but the patient had type 2 diabetes, the prediction would be deemed correct, even though in practice this would be a serious misdiagnosis. Ironically, closely related conditions may end up in different CCSR categories: “chronic kidney disease, stage 1” (*ICD-10* code: N181) is in the *GEN003* CCSR category, whereas “hypertensive chronic kidney disease with stage 1 through stage 4 chronic kidney disease, or unspecified chronic kidney disease” (*ICD-10* code: I129) is in the *CIR008* CCSR category. This means that we would penalize the LLM if it does not know that the chronic kidney disease is of hypertensive origin, even if it does not have access to the patient history proving so (note that patient blood pressure may appear normal in hypertensive kidney disease due to medication).

Our method uses a more subjective assessment, where we let the LLM agent conducting the evaluation decide whether the prediction is acceptable based on its similarity to the ground truth and given the available data. For example, mixing up type 1 and type 2 diabetes would be considered a miss if there is relevant antibody and C-peptide data. At the very least, the model should suggest a further C-peptide test (as instructed via the prompt in Sarvari et al [24]) if not already in the data, to confirm the diagnosis. Another advantage of our approach is that it makes the reported hit rate less data dependent by removing noninferable diagnoses. However, in an ideal case, complete and detailed patient electronic health record data are available from multiple hospitals, locations, and demographics to test the diagnostic ability of LLMs. While the hit rate of these LLMs on such datasets might be different, we would expect the relative rankings of these models to stay the same.

### Performance Interpretation

While the architectural details and training data of proprietary models, such as GPT-4 series and Claude Sonnet models, are not publicly disclosed, several factors may plausibly account for their superior diagnostic performance observed in our study. These models likely leverage more advanced architectures, employ larger parameter counts (eg, GPT-4 is estimated at 1.8T parameters according to industry reports), use more diverse training corpora, and benefit from sophisticated instruction tuning and reinforcement learning from human feedback [74]. Such attributes can enhance their ability to extract subtle clinical patterns, synthesize complex information from comorbid patient records, and generalize across diverse diagnostic categories. For example, larger model sizes and broader training data could result in a more robust internal medical knowledge base and improved reasoning capabilities, particularly when faced with ambiguous or incomplete clinical data. Additionally, ongoing improvements in prompt handling and context window size may enable these latest-generation LLMs to process longer, more complex patient summaries without losing track of key details, further supporting accurate diagnosis in comorbid cases. The observed differences may also reflect disparities in how LLMs were exposed to medical literature, clinical guidelines, and case data during training. If certain models receive more exposure to up-to-date or highly curated medical information, they may be better positioned to infer diagnoses based on subtle findings or atypical presentations. While OpenAI and Anthropic have not disclosed this information, Google has publicly stated that Gemini uses MoE (Mixture of Experts). In Gemini’s MoE architecture, the model dynamically routes each portion of the text input to a small set of specialized submodels (“experts”), each of which has developed unique capabilities during training. This specialization emerges naturally as the model learns to distribute different types of inputs, such as complex narratives, factual queries, and long-context reasoning, across the experts best suited to process them. As a result, the MoE approach enables Gemini to efficiently focus computational resources on the most relevant parts of the input, improving both quality and speed. This design boosts performance on large-scale language tasks, allowing the model to generalize better, follow prompts, and reason more deeply [75].

The top hits and misses (Table 5) show a similar pattern to information from the study by Sarvari et al [24]: highly prevalent and routinely documented conditions like diabetes, heart failure, or kidney disease are more likely to appear in clinical datasets and may thus be more reliably recognized by LLMs. At the same time, conditions like dehydration, hypotension, and hypoxemia often coexist with or are secondary to other critical illnesses. If not clearly distinguished in the record, an LLM may attribute findings to the primary diagnosis, missing the specific secondary issue. This challenge is particularly pronounced for electrolyte imbalances (eg, hypokalemia, hypernatremia, and hyponatremia) and disorders of osmolality, which often arise as secondary phenomena in critically ill or comorbid patients. In complex cases, both clinicians and LLMs may prioritize primary diagnoses (such as kidney failure and heart failure), while abnormalities in sodium, potassium, or osmolality are either not explicitly highlighted in the record or simply treated as laboratory abnormalities rather than standalone diagnoses. When these findings are not clearly distinguished, the model is more likely to miss them, either attributing the abnormality to the underlying primary disease or failing to identify the abnormality altogether due to a lack of explicit mention or reference range context. This ties back to the value of providing up-to-date reference range information to LLMs. One might provide only the most relevant sections of a guideline using RAG or input the entire guideline into the LLM’s context. Some of the latest models, such as Gemini 2.5 and GPT-4.1, now feature a 1 million token context window (roughly 1500 pages), and LLaMa 4 Scout provides an industry-leading 10 million context window [45], making it technically feasible to process entire medical guidelines as context. However, the use of RAG remains beneficial for reducing cost and focusing the model’s attention, thereby supporting more efficient and targeted diagnostic reasoning.

### Limitations

We would like to draw attention to the shortcomings of this study. First, we only considered a single dataset from a single hospital. Second, this dataset did not contain all the information that doctors normally use for diagnosing patients, resulting in the exclusion of some important diagnoses from the analysis as they were deemed noninferable. In fact, in practice, decision-making goes beyond text-based data from the electronic patient record, and without an AI system taking multimodal inputs sitting alongside a doctor as part of a proper hospital pilot, it will be very difficult to truly compare the diagnostic ability of LLMs to that of doctors. Third, in this study, we allowed LLMs to make many predictions; however, in practice, doctors may need to rely on a single diagnosis and treatment plan, which is their current best estimate. Fourth, this study did not consider images and only took natural language as an input; this is a crucial limitation, especially as the aforementioned studies indicated the shortcomings of GPT-4o in medical image analysis [28-30]. Fifth, this study did not assess biases in the predictions made by the different models, which would be an essential first step toward hospital deployment of LLMs. Readers looking to learn more about this topic are directed to the study by Zack et al [27]. Sixth, in this study, we only tested the performance of LLMs in the English language. While English is widely accepted as the international language of medicine [76], LLMs undoubtedly would need to speak multiple languages to truly help doctors around the world. Recent research suggests consistent diagnostic performance of GPT-4o across 9 different languages [77].

Lastly, it is important to keep in mind that the evaluation was done by an LLM and was not reviewed manually by a human, let alone a clinician. GPT-4 Turbo can be considered a lenient grader, and it classified multiple ground truth diagnoses as hits when noninferable would have been a better option (as pointed out by GPT-4.1; some examples are shown in Table 6). Additionally, GPT-4 Turbo occasionally misunderstood prompt terms such as “related.” For example, in admission ID 22780338, it incorrectly concluded that “chronic pulmonary disease” was not a new diagnosis because it was “related” to “chronic diastolic (congestive) heart failure,” failing to recognize that chronic obstructive pulmonary disease does not directly cause congestive heart failure. We also observed instances where GPT-4 Turbo misclassified historical diagnoses as “not new,” even when they were not predicted correctly. Furthermore, in some cases, the model did not follow the one-shot learning example provided in the evaluation prompt. Instead of offering a full rationale, it returned a 1-word answer to question 1. Since our automated evaluation relied on parsing model reasoning to distinguish between hits and *ICD* codes that are not true medical diagnoses, the behavior caused some nonmedical *ICD* codes (eg, unemployment) to be incorrectly marked as hits. All of these issues likely inflated the model’s hit rate. Future studies should consider using GPT-4.1 or similarly more accurate and conservative models for automated evaluation or should ideally include human expert review to validate the grading process [27,35].

## Abbreviations

AI: artificial intelligence
API: application programming interface
CCSR: Clinical Classifications Software Refined
ECG: electrocardiography
*ICD*: *International Classification of Diseases*
*ICD-10*: *International Classification of Diseases, 10th Revision*
LLM: large language model
MIMIC-IV: Medical Information Mart for Intensive Care-IV
MoE: Mixture of Experts
NEJM: *New England Journal of Medicine*
RAG: retrieval-augmented generation
